# Direct Observation of Photoinduced Charge Separation in Ruthenium Complex/Ni(OH)_2_ Nanoparticle Hybrid

**DOI:** 10.1038/srep18505

**Published:** 2015-12-17

**Authors:** Yu Tang, Brian Pattengale, John Ludwig, Abderrahman Atifi, Alexander V. Zinovev, Bin Dong, Qingyu Kong, Xiaobing Zuo, Xiaoyi Zhang, Jier Huang

**Affiliations:** 1 Department of Chemistry, Marquette University, Milwaukee, Wisconsin, 53201; 2 Material Science Division, Argonne National Laboratory, Argonne, Illinois, 60349; 3 X-ray Science Division, Argonne National Laboratory, Argonne, Illinois, 60349.

## Abstract

Ni(OH)_2_ have emerged as important functional materials for solar fuel conversion because of their potential as cost-effective bifunctional catalysts for both hydrogen and oxygen evolution reactions. However, their roles as photocatalysts in the photoinduced charge separation (CS) reactions remain unexplored. In this paper, we investigate the CS dynamics of a newly designed hybrid catalyst by integrating a Ru complex with Ni(OH)_2_ nanoparticles (NPs). Using time resolved X-ray absorption spectroscopy (XTA), we directly observed the formation of the reduced Ni metal site (~60 ps), unambiguously demonstrating CS process in the hybrid through ultrafast electron transfer from Ru complex to Ni(OH)_2_ NPs. Compared to the ultrafast CS process, the charge recombination in the hybrid is ultraslow (≫50 ns). These results not only suggest the possibility of developing Ni(OH)_2_ as solar fuel catalysts, but also represent the first time direct observation of efficient CS in a hybrid catalyst using XTA.

Generation of H_2_ from water by solar energy conversion is an attractive strategy to partially address the energy crisis and climate issues^1^. The overall process for water splitting includes two half-catalytic reactions, i. e. hydrogen (HER) and oxygen evolution reactions (OER)^2^,^3^,^4^. Each of the reactions requires a photocatalytic system that can efficiently integrate multiple components: a photosensitizer (PS) for light harvesting, a catalyst that can perform the appropriate half-reaction, and finally a reaction medium to allow charge flow from the excited PS to the catalyst and electron/hole acceptors^2^. Since the initial report of light-driven splitting of water^5^, extensive efforts have been devoted to develop such photocatalytic systems. One class that has been intensively studied uses the platinum group metals or metal oxides^1^ as catalysts. While these durable catalytic systems can efficiently catalyze HER or OER, their long term practicality is hampered by the scarcity and high cost of noble metals. As a result, various low-cost electrocatalysts such as MoS_2_^7^,^8^,^9^ and NiMo catalysts^10^,^1^1 for HER and CoPi^12^,^1^3 and metal oxides^14^,^1^5 for OER, have evolved during the past decade. However, most of these earth abundant catalysts are only active or stable in either acidic or basic electrolytes. Given that the overall water splitting requires that both HER and OER catalysts exist in the same electrolyte, it is crucial to develop bifunctional catalysts that can efficiently catalyze HER and OER in the same electrolyte.

Ni(OH)_2_ nanocrystals have emerged as solar fuel catalysts due to their low-cost and bifunctional nature^16^,^17^,^18^,^19^,^20^,^21^,^22^,^23^. OER can be efficiently driven using Ni(OH)_2_ nanocrystals as catalysts in alkaline electrolyte^16^,^1^7. Ni(OH)_2_ modified metal surfaces are also efficient HER catalysts with higher activities than bare metal catalysts^19^,^2^0. Recently, water photolysis with 12.3% efficiency was achieved using an Fe incorporated Ni(OH)_2_ foam electrode as a catalyst for both HER and OER^21^, evidently demonstrating the potential of Ni(OH)_2_ as a bifunctional photocatalyst for water splitting. Despite these studies, a fundamental understanding of the photoinduced charge separation (CS) and charge recombination (CR) dynamics in Ni(OH)_2_ based photocatalytic systems, the processes crucial for solar fuel generation, is still lacking.

In this work, we report a new hybrid photocatalytic system employing monodispersed Ni(OH)_2_ colloidal nanoparticles (NPs) as catalysts and RuN3 (cis-diisothiocyanato-bis(2,2’-bipyridyl-4,4’-dicarboxylic acid) ruthenium(II)) as a PS (Fig. 1a). We unambiguously demonstrated electron injection from excited RuN3 to Ni(OH)_2_ NPs using the combination X-ray (XTA) and optical transient absorption spectroscopy (OTA). RuN3 was chosen as a model PS because it is among the most efficient and robust molecular PS for light harvesting with well-known optical properties, and thus facilitated our photophysical studies. Moreover, RuN3 has –COOH groups which have extensively been used as linkers to bind dye molecules to the surface of metal oxides and can facilitate assembling on the surface of Ni(OH)_2_ NPs^24^,^2^5. The details of the synthesis of Ni(OH)_2_ NPs and RuN3/Ni(OH)_2_ hybrid are described in supplementary information (SI). The average diameter of Ni(OH)_2_ NPs measured by small-angle X-ray scattering (SAXS) is 4.42 ± 0.40 nm (Supplementary Fig. 1), in agreement with our TEM data (Supplementary Fig. 2).

## Results and Discussion

**Figure 1:**
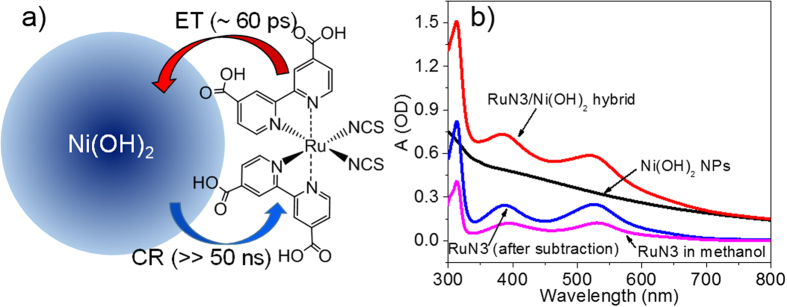
The design of hybrid catalysts. (**a**) Cartoon of charge separation and recombination dynamics in RuN3/Ni(OH)_2_ hybrid. (**b**) UV-visible absorption spectra of Ni(OH)_2_ NPs (black), RuN3/Ni(OH)_2_ hybrid (red), RuN3 obtained after subtracting the spectrum of Ni(OH)_2_ NPs from the spectrum of the hybrid (blue), and RuN3 in methanol (pink).

Figure 1b shows the UV-visible absorption spectra of Ni(OH)_2_ NPs and RuN3/Ni(OH)_2_ hybrid in toluene solution. The spectrum of Ni(OH)_2_ NPs shows a broad absorption in the whole UV-visible region, which can be attributed to the intra-3d transitions in Ni^2+^ ions^26^,^2^7. Compared to the spectrum of Ni(OH)_2_ NPs, the spectrum of RuN3/Ni(OH)_2_ hybrid shows two additional absorption bands at 385 and 535 nm, respectively. These two bands, consistent with those in RuN3 in methanol solution (pink line in Fig. 1b) can be assigned to the metal-to-ligand charge transfer (MLCT) bands of RuN3^25^,^2^8. Because RuN3 is not soluble in toluene, these additional absorption features in the spectrum of RuN3/Ni(OH)_2_ hybrid in toluene solution are due to the directly adsorbed RuN3 on the surface of Ni(OH)_2_ NPs.

**Figure 2:**
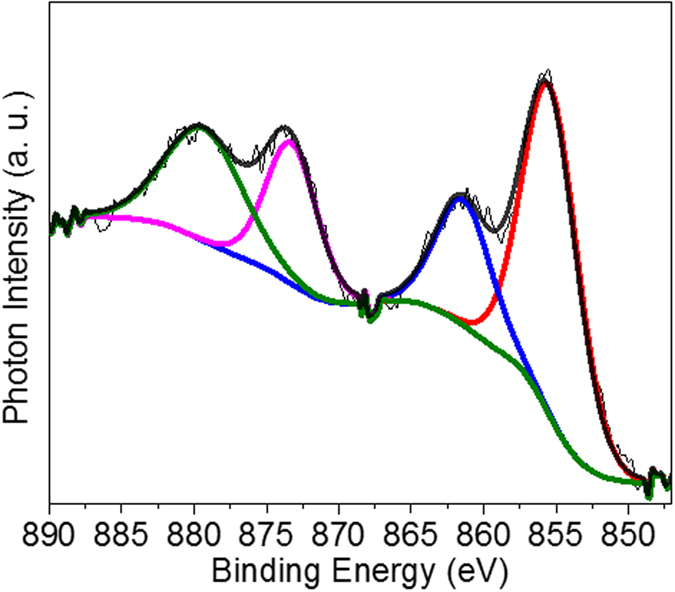
Chemical composition characterization. XPS spectrum of Ni(OH)_2_ NPs for binding energies within the range of 840–890 eV.

The chemical composition of the synthesized Ni(OH)_2_ NPs were analyzed by X-ray photoelectron spectroscopy (XPS) and steady state X-ray absorption spectroscopy (XAS). Figure 2 shows the high resolution XPS of Ni(OH)_2_ NPs at Ni 2p region (845–890 eV binding energy). The Ni 2p spectrum consists of two doublet of Ni 2p_3/2_ and Ni 2p_1/2_ transitions: Ni 2p_3/2_ main peak at 855.5 eV and its satellite at 861.5 eV, and Ni 2p_1/2_ main peak at 873.6 eV and its satellite at 879.7 eV. These results are consistent with the literature data of Ni(OH)_2_ and thus correspond to the presence of the nickel hydroxide^29^,^3^0. After subtracting the Shirley-type background, these peaks can be adequately fitted by four curve-fitting bands with Gaussian (50%)-Lorentzian (50%) profile. The five curve-fitting model using either pure Gaussian or Gaussian-Lorentzian function introduced significant noise, together with the fact that the sizes of the NPs (4.42 ± 0.40 nm in diameter) are small enough for XPS to penetrate, suggesting that the NPs are composed of Ni(OH)_2_.

**Figure 3:**
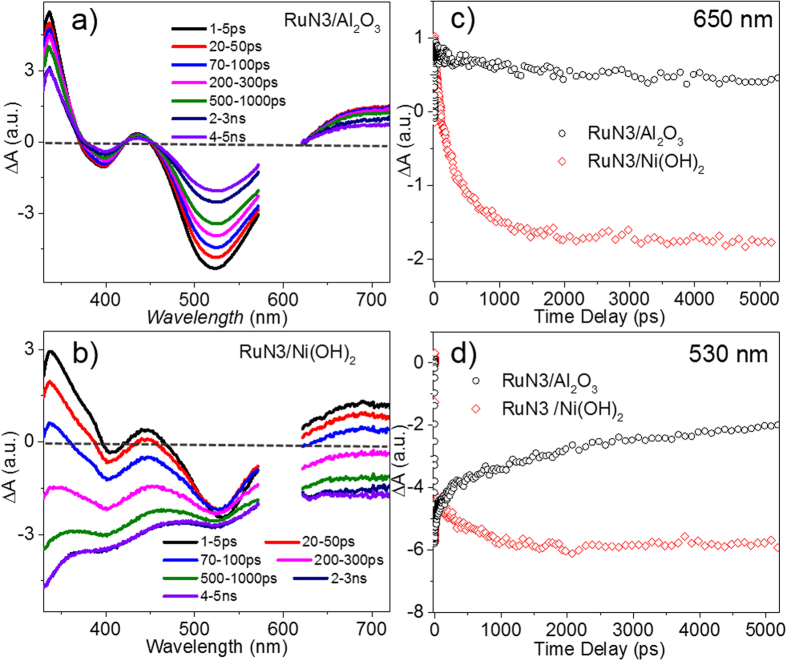
Transient absorption optical spectral features of the hybrid. Femtosecond OTA spectra of RuN3/Al_2_O_3_ (**a**) and RuN3/Ni(OH)_2_ hybrid (**b**). Comparison of the femtosecond OTA kinetics of RuN3/Al_2_O_3_ and RuN3/Ni(OH)_2_ hybrid at 650 nm (**c**) and 530 nm (**d**).

The CS dynamics in the RuN3/Ni(OH)_2_ hybrid in toluene were investigated by femtosecond OTA (fs-OTA). Figure 3a shows the fs-OTA spectra of RuN3/Al_2_O_3_ hybrid system, which represents a non-electron injecting model to reveal the intrinsic excited state (ES) dynamics of RuN3 on the surface of NPs. The spectra of RuN3/Al_2_O_3_ hybrid consist of two negative bands centered at 385 and 535 nm, and two broad positive absorption bands centered at 337 and 680 nm. The two negative bands are consistent with the ground state (GS) MLCT absorption bands and are thus assigned to the GS bleach of RuN3. The two absorption bands centered at 337 and 680nm, analogous to literature data^25^,^2^8, can be assigned to the ligand localized 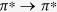 transition and ligand-to-metal charge transfer (LMCT) of RuN3 ES, respectively.

Compared to the fs-OTA spectra of RuN3/Al_2_O_3_, the spectra of RuN3/Ni(OH)_2_ hybrid (Fig. 3b) are drastically different. Although the initial spectra (1–5 ps) of RuN3/Ni(OH)_2_ hybrid resemble the spectra of RuN3/Al_2_O_3_, the broad ES bands of the former decay much faster within 5 ns time window, suggesting enhanced ES decay dynamics in RuN3/Ni(OH)_2_ hybrid. Furthermore, the spectra of RuN3/Ni(OH)_2_ hybrid evolve to a broad bleach band within the whole spectral region after the ES absorption signal decays substantially (>100 ps). These features can be further seen by comparing the kinetic traces of RuN3/Ni(OH)_2_ hybrid and RuN3/Al_2_O_3_ at 650 nm (Fig. 3c) and 530 nm (Fig. 3d) which correspond to the ES absorption and GS bleach of RuN3, respectively. Compared to the ES decay kinetics of RuN3/Al_2_O_3_ due to intrinsic recovery of GS from ES, the kinetic trace of RuN3/Ni(OH)_2_ hybrid at 650 nm quickly decays to negative after 100 ps, suggesting that the ES decay of RuN3 in RuN3/Ni(OH)_2_ hybrid is accompanied by the formation of negative signal. The formation of negative features were also observed at 530 nm, where the amplitude of the fs-OTA signal grows to more negative in RuN3/Ni(OH)_2_ hybrid instead of GS bleach recovery in RuN3/Al_2_O_3_. We can exclude the contribution of GS bleach of RuN3 to the negative signal at >650 nm where RuN3 has negligible GS absorption. We can also rule out the possibility that the negative signals are due to the direct excitation of Ni(OH)_2_ NPs, because negligible fs-OTA signals of Ni(OH)_2_ NPs were observed under the same experimental conditions (Supplementary Fig. 3). Indeed, this negative signal resembles the GS absorption of RuN3/Ni(OH)_2_ hybrid and thus can be assigned to the combination of GS bleach of RuN3 and Ni(OH)_2_ NPs.

**Figure 4:**
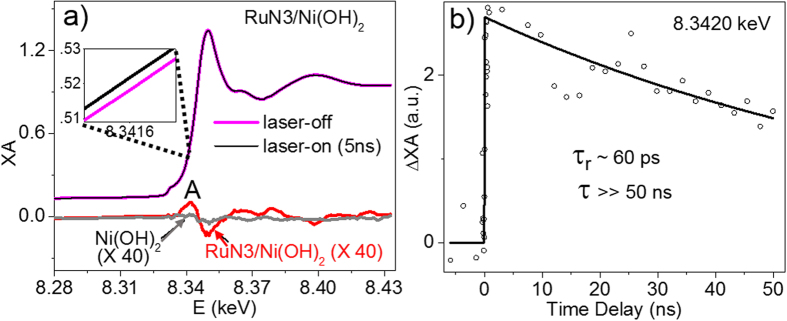
Transient X-ray absorption spectra at Ni K-edge. (**a**) XANES spectra of RuN3/Ni(OH)_2_ hybrid at Ni K edge before (laser-off) and after (laser-on) laser excitation. Inset shows the spectra at 8.3415–8.3417 keV range. The difference spectra obtained from subtracting the laser-off XANES spectrum from laser-on spectrum (5 ns) for RuN3/Ni(OH)_2_ hybrid (red solid line) and free Ni(OH)_2_ NPs (gray solid line) are presented below the XANES spectra. (**b**) The time dependence of the transient signal at 8.3420 keV as a function of delay time.

The formation of GS bleach of Ni(OH)_2_ NPs, along with the faster RuN3 ES quenching in RuN3/Ni(OH)_2_ hybrid, can arise from two possible pathways: electron transfer (ET) and hole transfer (HT). Although these processes result in different transient products (i.e. oxidized RuN3 and reduced Ni(OH)_2_ NPs for ET and reduced RuN3 and oxidized Ni(OH)_2_ NPs for HT), it is challenging to identify them using OTA because 1) metal centered states are often optically silent and 2) there are significant spectral overlaps between RuN3 and Ni(OH)_2_ NPs. To overcome this problem, element specific XTA, which allows us to selectively probe the specific metal center, was used to track the electron density change at Ni center during the photoinduced process in RuN3/Ni(OH)_2_ hybrid. Figure 4a shows XANES spectra of RuN3/Ni(OH)_2_ hybrid before (pink line) and 5 ns after 527 nm excitation (black line). The transient signal due to laser excitation was clearly observed in the expanded XANES spectra at 8.3415–8.3417 keV (inset) and the difference XANES spectrum (red solid line) after subtracting the laser-off spectrum from the laser-on spectrum. The positive feature at 8.3420 keV in the difference spectrum indicates that the edge of Ni center shifts to lower energy due to photoexcitation of RuN3 in the RuN3/Ni(OH)_2_ hybrid. This feature was not observed in the difference spectrum of Ni(OH)_2_ NPs alone (gray solid line), ruling out the possibility that the observed Ni edge shift is due to direct excitation of Ni(OH)_2_ NPs. These results unambiguously determine the ET process from excited RuN3 to Ni(OH)_2_ NPs.

In order to determine the ET rate, the intensity of the positive feature at 8.3420 keV in the difference spectrum of RuN3/Ni(OH)_2_ hybrid as a function of delay time was collected. As shown in Fig. 4b, the kinetics shows an instant rising followed by a slow decay. Fitting the kinetics data with an exponential decay function yields a rising component (τ_r_ ~ 60 ps, within 80 ps X-ray pulse duration) and a decay component (τ  ≫ 50 ns), corresponding to ET and CR times, respectively. The much slower CR compared to ET process suggests the potential application of Ni(OH)_2_ NPs as solar fuel catalysts.

Although ET and CR processes in RuN3/Ni(OH)_2_ hybrid were confirmed by XTA, the possibility of energy transfer (ENT) needs to be considered due to spectral overlap between RuN3 emission and Ni(OH)_2_ absorption (Supplementary Fig. 4). In principle, ENT efficiency can be estimated by measuring the emission of Ni(OH)_2_ NPs. Unfortunately, due to the extremely low emission quantum yield of Ni(OH)_2_ NPs, we are unable to measure the ENT efficiency by measuring the emission of Ni(OH)_2_ NPs. As shown in equation (1) and (2),


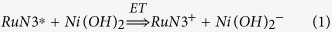



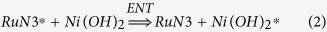


ENT and ET are two competitive pathways for quenching ES of RuN3. Different from the OTA spectral features of ET process where fast ES quenching is accompanied by long-lived GS, the expected OTA spectral features of ENT is characteristic of simultaneously occurring of fast ES decay and GS recovery. Given the known ultrafast ET rate determined by XTA (~ 60 ps), if ENT is a competitive pathway to ET, an identical ultrafast GS bleach recovery would occur in the spectral range of 500-550nm in OTA spectra of the hybrid, which however was not observed (Fig. 3b,d). These results suggest that ENT is not the dominating process for RuN3 ES quenching and efficient ET occurs from excited RuN3 to Ni(OH)_2_ NPs.

In summary, we have examined the photoinduced CS dynamics in a newly designed RuN3/Ni(OH)_2_ hybrid photocatalytic system using the combination of OTA and XTA. Upon photoexcitation of RuN3, ultrafast electron injection from the excited RuN3 to Ni(OH)_2_ NPs was observed, which is followed by ultraslow CR. These findings not only suggest the potential application of Ni(OH)_2_ NPs as photocatalysts for solar fuel conversion, but also demonstrate the superior capability of XTA in directly capturing the transient charge separated state in hybrid solar fuel catalysts.

## Additional Information

**How to cite this article**: Tang, Y. *et al.* Direct Observation of Photoinduced Charge Separation in Ruthenium Complex/Ni(OH)^2^ Nanoparticle Hybrid. *Sci. Rep.* **5**, 18505; doi: 10.1038/srep18505 (2015).

## Supplementary Material

Supplementary Information
