# Supplementary material for: Direct Observation of Photoinduced Charge Separation in Ruthenium Complex/Ni(OH)2 Nanoparticle Hybrid
Source: Sci Rep. 2015 Dec 17;5:18505. doi: 10.1038/srep18505 (PMC4682087; doi:10.1038/srep18505)
Supplement: Supplementary Information [file srep18505-s1.pdf]

Supporting Information

Direct Observation of Photoinduced Charge Separation in Ruthenium Complex/$Ni(OH)_2$ Nanoparticle Hybrid

Yu Tang,[a] Brian Pattengale,[a] John Ludwig,[a] Abderrahman Atifi,[a] Alexander V. Zinovev,[b] Bin Dong,[a] Qingyu Kong,[c] Xiaobin Zuo,[c] Xiaoyi Zhang,[c] Jier Huang[a]**

[a]*Department of Chemistry, Marquette University, Milwaukee, Wisconsin, 53201*

[b]Material Science Division and [c]X-ray Science Division, Argonne National Laboratory, Argonne, Illinois, 60349

1. General Materials. The starting chemicals were purchased from VWR and used as received. RuN3 (cis-diisothiocyanato-bis(2,2'-bipyridyl-4,4'-dicarboxylic acid) ruthenium(II)) was purchased from Solaronix. UV/visible spectra were measured on Agilent 8453 with 2 mm quartz cell.

2. The synthesis of $Ni(OH)_2$ NPs. $Ni(OH)_2$ NPs were synthesized according to the established literature procedure[1] with minor revision. Briefly, $Ni(acac)_2$ (257 mg,1 mmol) was dissolved in 15 mL oleylamine (OAm) and 0.32 mL (1 mmol) oleic acid (OA). The mixture was heated to 110 °C and kept at 110 °C for one hour under N_2 flow. After the mixture was cooled down to 90 °C, Borane tert-butylamine complex (BTBC) solution (175 mg in 2 mL of OAm) was injected into the solution quickly. The solution was kept at 90 °C for 60 min and then cooled down to room temperature. The desired $Ni(OH)_2$ NPs were precipitated out from the mixture by adding a large amount of ethanol. The obtained $Ni(OH)_2$ NPs can be redispersed in organic solvents such as hexane, toluene or benzene etc.

3. The synthesis of RuN3/$Ni(OH)_2$ hybrid. The preparation of RuN3/$Ni(OH)_2$ hybrid follows the procedure for semiconductor nanoparticle/molecular adsorbate hybrid reported elsewhere.[2,3] Briefly, 20 μL saturated RuN3 solution in methanol are added to 10 mL $Ni(OH)_2$ NPs in toluene solution. The mixture is sonicated and filtered to remove undissolved RuN3. Because RuN3 is not soluble in toluene, all dissolved RuN3 in the mixture solution is believed to be bound to the surface of $Ni(OH)_2$ NPs.[3-5] The presence of RuN3 on the surface of $Ni(OH)_2$ NPs is confirmed by monitoring RuN3 absorption in the hybrid using UV-visible absorption spectroscopy.

4. The synthesis of Al_2O_3 NPs. A solvothermal method was utilized from a combination of literature procedures.[6-9] In a typical synthesis, 3.75 g of $Al(NO_3)_3{\cdot}9H_2O$ and 0.6 g of NaOH were dissolved in 70mL of 50% ethanol separately. The combined mixture was stirred for 4 hours at room temperature followed by the addition of 5 mL of NH_4OH. The resulting cloudy white hydroxide precursor precipitant was collected via centrifugation and rinsed with water. The obtained gel was then autoclaved in a Parr Teflon-lined autoclave at 200°C for 16 hours.

5. Femtosecond absorption spectroscopy is available in our research lab. The spectrometer is based on a regenerative amplified Ti-Sapphire laser system (Solstice, 800nm, < 100 fs FWHM, 3.5 mJ/pulse, 1 KHz repetition rate). The tunable pump is generated in TOPAS which has output with tunable wavelength ranging from 254 nm to 960 nm. The tunable UV-visible probe pulses are generated by while light generation in a CaF_2 window (330-720 nm) on a translation stage. The femtosecond transient absorption is performed in Helios ultrafast spectrometer (Ultrafast Systems LLC). The energy of the 600 nm pump pulse used for the measurements was 454 nJ. The sample cuvette path length was 2 mm.

6. X-ray Photoelectron Spectroscopy was performed with the use of XPS/UPS instrument at Material Science Division of Argonne National Laboratory. This home-build instrument is

equipped with Perkin-Elmer X-ray dual anode Φ04-500 gun (MgK_α line, $h\omega$=1253.6 eV, 300W output power), hemispherical energy analyzer (VSW HA100) and 16-anode detector. The total energy resolution of the instrument in given operating mode was $\Delta\varepsilon$=0.93 eV. Energy calibration of the spectrometer is performed in routing manner with the use of freshly deposited Au standard. The sample (NPs solution in toluene) was deposited onto Si wafer substrate (0.4 mm thickness), dried off and place into vacuum chamber with ultimate residual gas pressure about $6x10^{-8}$ Pa. Obtained XPS data were processed with using Casa XPS software.

7. Time resolved X-ray absorption spectroscopy measurements were performed at the beamline 11ID-D of Advanced Photon Source (APS) at Argonne National Laboratory. The laser pump was based on a Nd:YLF regenerative amplified laser (1053 nm, 1.6 kHz repetition rate, 5 ps FWHM). The pump wavelength at 527 nm was obtained from the output of second harmonic generation. The X-ray pulse with 80 ps FWHM width at 6.5 MHz repetition rate was used as the probe. The laser pump and X-ray probe intersect at a flowing sample stream with 550 μm in diameter. The X-ray fluorescence signals were collected at 90˚ angle on both sides of the incident X-ray beam by two avalanche photodiodes (APDs). A soller slits/Co filter combination, which was custom-designed for the specific sample chamber configuration and the distance between the sample and the detector, was inserted between the sample stream and the APD detectors. The emitted Ni X-ray fluorescence collected at 5 ns after the laser pump pulse excitation of RuN3/$Ni(OH)_2$ hybrid was used to build the spectrum of reduced state of $Ni(OH)_2$ NPs in advanced photon source standard operating mode. The Ni fluorescence resulting from averaging the previous 50 round trips in the storage ring prior to the laser pulse were used to construct the ground state spectrum of $Ni(OH)_2$ NPs.

8. Size and Distribution by Small Angle X-ray Scattering. To determine the size and distribution of $Ni(OH)_2$ NPs, we performed solution small angle x-ray scattering (SAXS) at beamline 12ID-B of the Advanced Photon Source at Argonne National Laboratory. The SAXS data are displayed in Figure S1 (red curve). The data fitting was performed over the q range of 0.014-0.4 Å^{-1}, where q is scattering momentum transfer, $q = \frac{4\pi sin\theta}{\lambda}$. θ is the Bragg angle and λ is the wavelength the incident x-ray, which is set as 0.886 Å for the measurement.

The particles were approximated with spherical shape with homogeneous electron density (ρ) and a form factor of a spherical object, P (q,R), was used in the fitting, $P(q,R) = \rho \frac{4\pi R^3}{3}\left[\frac{3(\sin(qR) - qRcos(qR)}{(qR)^3}\right]^2$, where R is the radius of the sphere.[10] SAXS measures millions of nanoparticles in the x-ray beam path. The nanoparticles were assumed to follow log normal distribution with respect to particle radii. The log normal distribution function is expressed as:

$$n(R, R_0, \sigma) = \frac{1}{R\sigma\sqrt{2\pi}} \exp(\frac{-(\ln(R/R_0))^2}{2\sigma^2}),$$

where R0 is the maximum population position and σ describes the distribution width. The total x-ray scattering from the particles in the x-ray beam can be written as:

$$I(q) = \int P(q,R)n(R,R_0,\sigma)dR. \quad \text{(Eq. 1)}$$

SAXS data fitting using Eq. 1 was performed in Matlab by minimizing the following penalty function, χ^2:

$$\chi^2 = \sum_j \left(\frac{I_{exp}(q_j) - \alpha I_{calc}(q_j)}{s(q_j)}\right)^2$$

where $I_{exp}(q_j)$ and $I_{calc}(q_j)$ are experimental data and scattering intensity calculated from Eq1, respectively, α is a scaling factor, and $s(q_j)$ is the value of experimental error at q_j. A good fit was achieved in the whole range (Figure 1a) and a relatively narrow distribution was yielded from the

fitting (Figure S1b). The overall radius obtained from the fitting is 22.1 ± 4.0 Å, which is consistent with our TEM results (Figure 2).

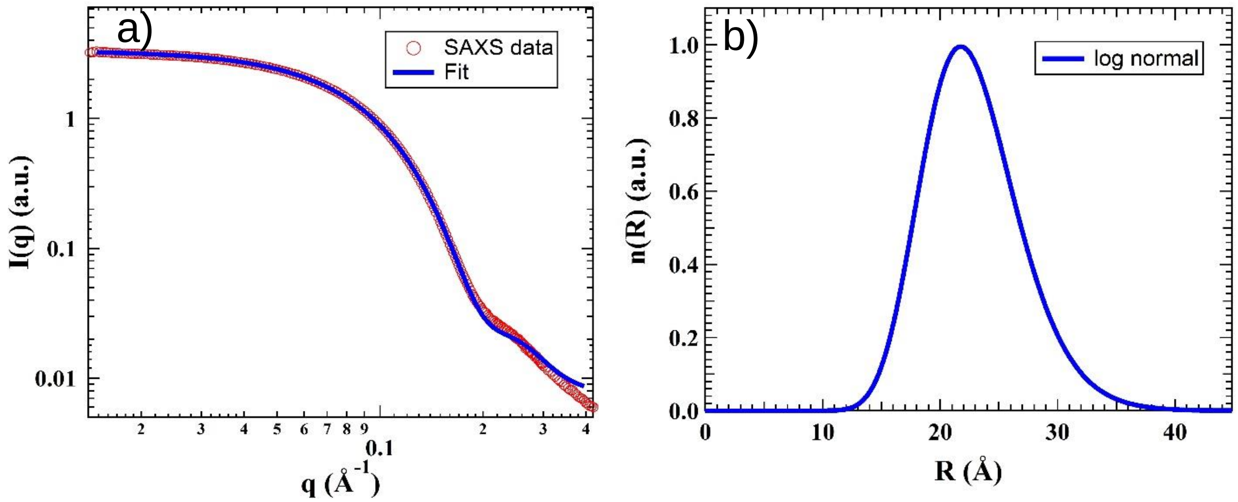

Figure 1. SAXS data and size distribution fitting for $Ni(OH)_2$ NPs. (a) SAXS data (red) and fit (blue) using log norm distribution. (b) Particle radius distribution yielded from the SAXS data fit in (a) under spherical shape assumption. Fitting parameters are R_0= 21.8 Å, the most populated radius; σ=0.181; and the overall size is 22.1 ± 4.0 Å.

9. Transmission Electron Microscopy (TEM). TEM images were taken on a JEOL JEM-2100F microscope operating at a voltage of 200 kV.

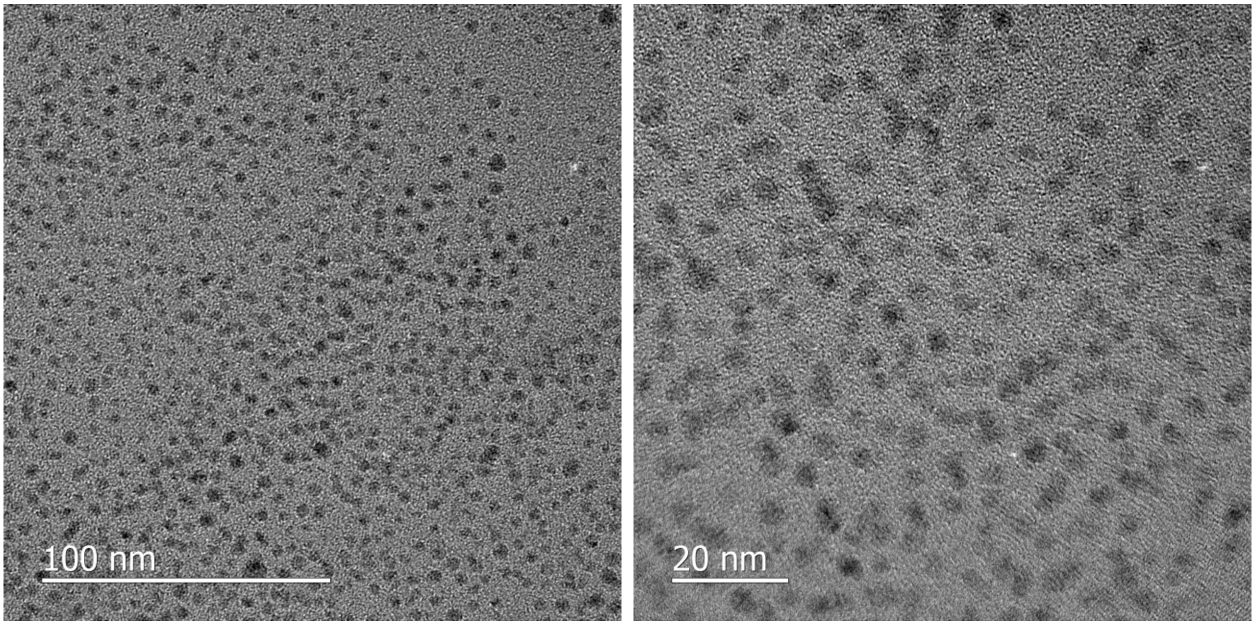

Figure 2. TEM images of $Ni(OH)_2$ NPs.

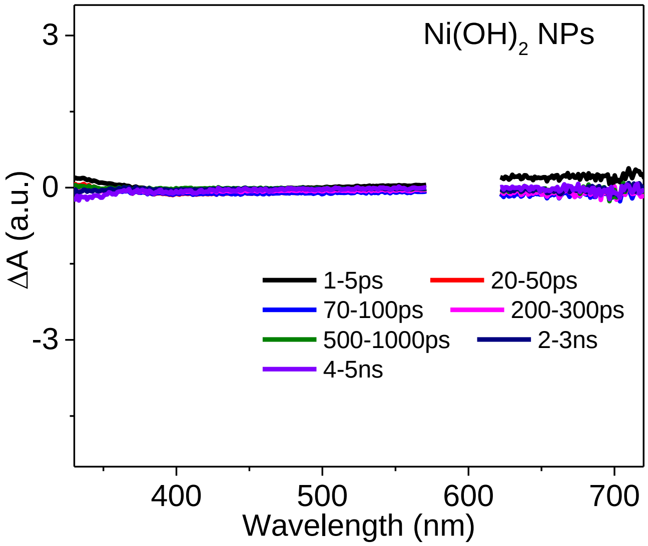

Figure 3. Femtosecond OTA spectra of $Ni(OH)_2$ NPs in toluene solution after 600 nm excitation.

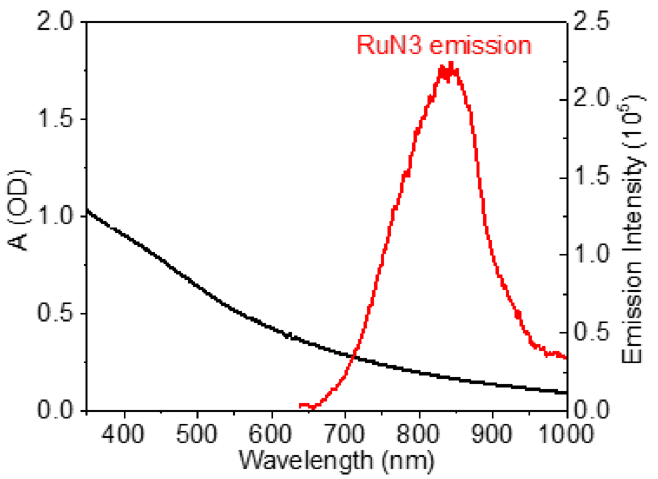

Figure 4. The emission spectrum of RuN3 in methanol (red) after 530 nm excitation and the absorption spectrum of $Ni(OH)_2$ NPs in toluene.

Reference

(1) Metin, O.; Mazumder, V.; Ozkar, S.; Sun, S. S. *J. Am. Chem. Soc.* **2010**, *132*, 1468.

(2) Huang, J.; Huang, Z.; Yang, Y.; Zhu, H.; Lian, T. *J. Am. Chem. Soc.*, **2010**, *132*, 4858.

(3) Huang, J.; Mulfort, K. L.; Du, P.; Chen, L. X. *J. Am. Chem. Soc.* **2012**, *134*, 16472.

(4) Tachibana, Y., Moser, J. E.; Gratzel, M.; Klug, D. R.; Durrant, J. R. 1996, *J. Phys. Chem.* **1996**, *100*, 20056.

(5) Zhang, J.; Gayatri, N.; Huang, Z.; Kokhan O.; Zhang, X.; Wu, Y. *J. Phys. Chem. C* **2012**, *116*, 16854.

(6) Carnes, C. L.; Stipp, J.; Klabunde, K. J. *Langmuir* **2002**, *18*, 1352.

(7) Yao, N.; Xiong, G. X.; Zhang, Y. H.; He, M. Y.; Yang, W. S. *Catal. Today* **2001**, *68*, 97.

(8) Dudkin, B. N.; Krivoshapkin, P. V.; Luksha, V. G. *Colloid. J.* **2006**, *68*, 40.

(9) Hayashi, H.; Hakuta, Y. *Materials* **2010**, *3*, 3794.

(10) Guinier, A.; G. Fournet, "Small-Angle Scattering of X-Rays", John Wiley and Sons, New York, (1955).
